# Polyphenolic Extracts from Spent Coffee Grounds Prevent H_2_O_2_-Induced Oxidative Stress in *Centropomus viridis* Brain Cells

**DOI:** 10.3390/molecules26206195

**Published:** 2021-10-14

**Authors:** Nayely Leyva-López, Melissa Peraza-Arias, Anaguiven Avalos-Soriano, Crisantema Hernández, Cynthia E. Lizárraga-Velázquez, J. Basilio Heredia

**Affiliations:** 1 Centro de Investigación en Alimentación y Desarrollo, A.C., Av. Sábalo Cerritos S/N, Mazatlán 82112, Sinaloa, Mexico; melissa.peraza.mc18@estudiantes.ciad.mx (M.P.-A.); anaguiven.avalos@ciad.mx (A.A.-S.); 2 Cátedras CONACYT-Centro de Investigación en Alimentación y Desarrollo, A.C., Av. Sábalo Cerritos S/N, Mazatlán 82112, Sinaloa, Mexico; 3 Centro de Investigación en Alimentación y Desarrollo, A.C., Carretera a Eldorado Km. 5.5, Col. Campo El Diez, Culiacán 80110, Sinaloa, Mexico; jbheredia@ciad.mx

**Keywords:** antioxidant capacity, catalase, superoxide dismutase, lipid peroxidation, white snook

## Abstract

Oxidative stress in aquatic organisms might suppress the immune system and propagate infectious diseases. This study aimed to investigate the protective effect of polyphenolic extracts from spent coffee grounds (SCG) against oxidative stress, induced by H_2_O_2_, in *C. viridis* brain cells, through an in vitro model. Hydrophilic extracts from SCG are rich in quinic, ferulic and caffeic acids and showed antioxidant capacity in DPPH, ORAC and FRAP assays. Furthermore, pretreatment of *C. viridis* brain cells with the polyphenolic extracts from SCG (230 and 460 µg/mL) for 24 h prior to 100 µM H_2_O_2_ exposure (1 h) significantly increased antioxidant enzymes activity (superoxide dismutase and catalase) and reduced lipid peroxidation (measured by MDA levels). These results suggest that polyphenols found in SCG extracts exert an antioxidative protective effect against oxidative stress in *C. viridis* brain cells by stimulating the activity of SOD and CAT.

## 1. Introduction

Aquaculture is one of the fastest-growing industries and it is becoming one of the main sources of food production worldwide [1]. It has been reported that aquaculture contributes around half of the fish production destined for human consumption [2]. Among cultured organisms, marine and freshwater fish, as well as crustaceans, are the most wanted. Due to the fast-growing and high demand for aquaculture products, fish farming has been intensified, which means more organisms are produced in smaller spaces. Furthermore, other factors, such as unbalanced aqua feeds, poor water quality, fluctuations in temperature, salinity, pH and oxygen availability in the growing medium, might generate oxidative stress in fish and influence their responses to the environmental changes, suppress their immune system and negatively affect their health [3].

Oxidative stress is a cellular condition caused by an uncontrolled increase in the production of reactive oxygen species (ROS; e.g., O_2_^−^, OH^−^ and H_2_O_2_), and the failure of the antioxidant enzymatic defense [e.g., superoxide dismutase (SOD), catalase (CAT) and glutathione peroxidase (GPx)], to neutralize them [4]. The excess of ROS creates a cellular oxidative environment and could potentially damage essential biomolecules. For instance, the excess promotes lipid peroxidation of the cellular membranes, enzymatic inactivation, damage in the DNA and homeostasis imbalance, among other factors [5].

Sometimes, the organisms’ cells might not be capable of neutralizing ROS, so the use of exogenous antioxidative components can help to counteract oxidative stress. Polyphenolic compounds from vegetable wastes are a very well-studied group of bioactive compounds that have demonstrated antioxidant properties [6], either by directly donating electrons/protons to stabilize ROS or by enhancing the activity of antioxidant enzymes, such as SOD and CAT [7]. Recent studies have demonstrated that polyphenols can reduce induced oxidative stress in pheochromocytoma (PC12) cells by lowering the levels of malondialdehyde (MDA), an oxidative stress marker, and by improving the activity of antioxidant enzymes, such as SOD and CAT [8,9]. Nevertheless, most of these studies have been carried out in mammal cells and research around the potential antioxidant and protective effect of polyphenols against induced oxidative stress in fish cells is very scarce [10].

Spent coffee grounds (SCG), the solid residue of coffee generated during the brewing process, is a rich source of antioxidant polyphenols, which are the main contributors to the antioxidant activity of coffee brews and their by-products [11]. Chlorogenic acids, the most abundant polyphenols in coffee, are esters of quinic acid bound to hydroxycinnamic acids, such as caffeic, ferulic and *p*-coumaric acids, to form a variety of conjugated compounds known as caffeoylquinic, feruloylquinic and *p*-coumaroylquinic acids [12,13]. Therefore, the use of polyphenolic compounds from SCG might be recommended to increase the antioxidant potential in any given model.

It has been demonstrated that fish species exposed to induced stress present oxidative damage in the brain [14,15]. The brain needs a high and constant oxygen supply to cover its energy needs, it produces more ROS per gram of tissue than any other organ in the body and it is highly vulnerable to radical attack and oxidative stress [16]. In this study, a primary culture of white snook *Centropomus viridis* brain cells was used as a model for studying the protective effects of polyphenolic extracts from SCG against oxidative stress. To determine the protective effect of polyphenolic extracts from SCG on oxidative stress, we successfully established and characterized a marine fish brain cell line, white snook *C. viridis*. *C. viridis* is a carnivorous brackish-water-based fish species. This species, which is distributed in the tropical regions of the eastern Pacific Ocean, and possess a high potential for intensive commercial farming [17].

The present study aimed to evaluate the antioxidant effect of polyphenolic extracts from spent coffee grounds against H_2_O_2_-induced oxidative stress in *C. viridis* brain cells.

## 2. Results

### 2.1. Phenolic Compounds and Antioxidant Capacity of Polyphenolic Extracts from SCG

The total phenolic content (TPC), of the SCG extract, measured by the Folin–Ciocalteu method, was 892.67 ± 59.12 mg GAE/100 g dry weight. The main compound present in the SCG extracts was quinic acid, which contributed to the 73.59% ± 5.37% of the extract’s TPC, followed by ferulic and caffeic acids (Table 1).

The antioxidant capacity of the polyphenolic extracts from SCG measured using the DPPH, FRAP and ORAC assays is shown in Table 2.

### 2.2. Non-Toxic Concentrations of Polyphenolic Extracts from SCG on C. viridis Brain Cells

**Figure 1:**
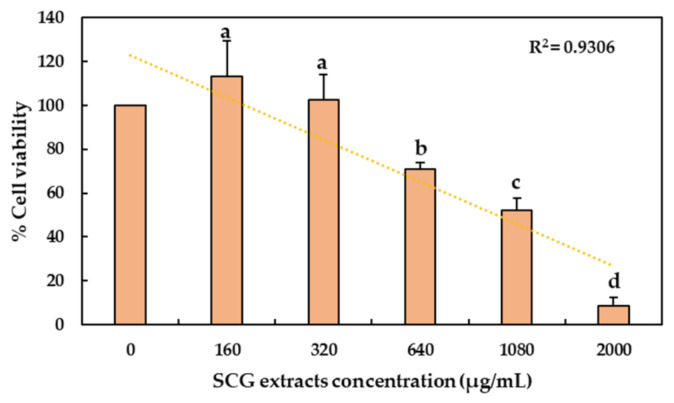
Effect of polyphenolic extracts from SCG on cell viability of *C. viridis* brain cells. Data are mean ± SD of three separate trials. Each treatment had three replicates (wells). Data are presented as the relative percentage of zero. Different letters (a, b, c, d) indicate significant differences between concentrations (*p* < 0.05).

To define the non-toxic concentrations of polyphenolic extracts from SCG on the *C. viridis* brain cells, the MTT cytotoxicity assay was performed. The highest concentrations tested, 2000, 1080 and 640 µg/mL, significantly decreased cell proliferation under 80% (*p* < 0.05). In contrast, lower concentrations did not affect cell viability when compared with the control (zero), without polyphenolic extracts (Figure 1). The maximum toxic concentration of polyphenolic extracts from SCG that sustains 80% of cell viability was calculated using linear regression, (y = −39.564x + 323.2, R^2^ = 0.9306) [18,19], and the result was 467.31 µg/mL. These data show that polyphenolic extracts from SCG could be used without cytotoxic effects at concentrations under 467.31 µg/mL.

### 2.3. Protective Effect of Polyphenolic Extracts from SCG against H_2_O_2_-Induced Oxidative Stress

**Figure 2:**
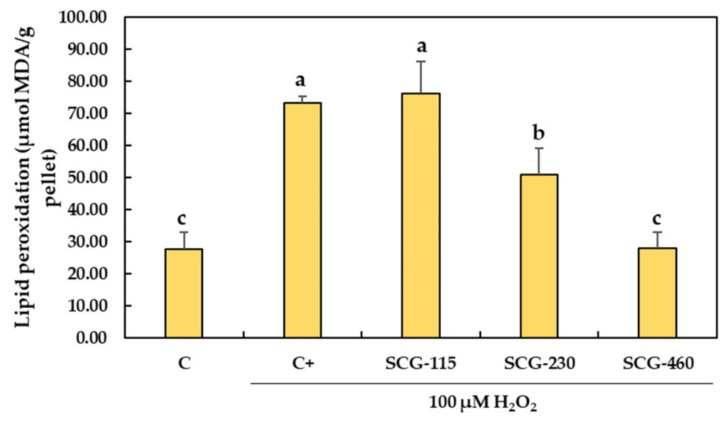
Effect of polyphenolic extracts from SCG on the lipid peroxidation in *C. viridis* brain cells under H_2_O_2_-induced oxidative stress. Cells were pretreated with the SCG extracts (115–460 µg/mL) for 24 h and then incubated in the presence of 100 µM H_2_O_2_ for 1 h. C: control, non-treated cells; C+: cells treated with 100 µM H_2_O_2_ alone; SCG: cells pretreated with the spent coffee ground polyphenolic extracts at 115, 230 and 460 µg/mL, and treated with 100 µM H_2_O_2_. Data are presented as means ± SD (*n* = 3). Different letters (a, b, c) indicate significant differences between the treatments (*p* < 0.05).

Lipid peroxidation is one of the most broadly studied indicators of oxygen toxicity in the biological systems. In this study, the MDA levels were used as a biomarker of lipid peroxidation, reflecting the oxidative stress in the *C. viridis* brain cells. A significant increase (*p* < 0.05) by 165.87% ± 7.55% of the MDA level compared with the control group (C) was observed in the *C. viridis* brain cells when exposed to H_2_O_2_ (100 µM) (Figure 2). Nevertheless, when cells were pretreated with the polyphenolic extracts from SCG at 230 µg/mL and 460 µg/mL, the MDA levels were reduced to 69.55% ± 11.24% and 39.66% ± 9.11%, respectively, as compared with the brain cells treated with H_2_O_2_ (*p* < 0.05). Furthermore, pretreatment of *C. viridis* cells with SCG-460 restored the MDA levels at the same level as in the non-exposed control group.

**Figure 3:**
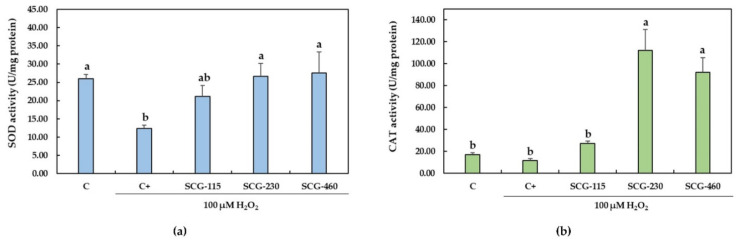
Effect of polyphenolic extracts from SCG on the (**a**) SOD and (**b**) CAT activities in *C. viridis* brain cells under H_2_O_2_-induced oxidative stress. Cells were pretreated with the SCG extracts (115–460 µg/mL) for 24 h and then incubated in the presence of 100 µM H_2_O_2_ for 1 h. C: control, non-treated cells; C +: cells treated with 100 µM H_2_O_2_ alone; SCG: cells pretreated with the spent coffee ground polyphenolic extracts at 115, 230 and 460 µg/mL, and treated with 100 µM H_2_O_2_. Data are presented as means ± SD (*n* = 3). Different letters (a, b, ab) indicate significant differences between the treatments (*p* < 0.05).

Antioxidant enzymes, such as SOD and CAT, are the primary line of defense of the living organisms against oxidative damage. Exposure of *C. viridis* brain cells to H_2_O_2_ (100 µM) significantly (*p* < 0.05) reduced the activity of SOD to 47.92% ± 3.18% as compared with the control (C) group (Figure 3a). Pretreatment with the polyphenolic extracts from SCG at 230 µg/mL and 460 µg/mL significantly reduced (*p* < 0.05) the decrease in SOD activity induced by the H_2_O_2_. CAT activity was not significantly affected by the exposure of cells to H_2_O_2_ (100 µM) (Figure 3b). Nevertheless, a reduction in CAT activity was observed by 30.53% ± 8.73% compared with the C group. The pretreatment of cells with SCG-230 and SCG-460 significantly increased the activity of CAT.

## 3. Discussion

In this original manuscript, the antioxidant capacity, in terms of radical scavenging activity, and the non-cytotoxic and protective effects of the polyphenolic extracts from spent coffee grounds against H_2_O_2_-induced oxidative stress in *C. viridis* brain cells have been demonstrated.

Aquatic organisms might be exposed to oxidative stress due to intensive farming conditions, such as low-quality nutrition of aquafeeds, scarce water quality and drastic changes in temperature, pH or oxygen availability in the growing medium [20]. The oxidative damage in cells is associated, in fish, with more susceptible organisms to the attack of pathogens and the spreading of infectious diseases [21]. Many researchers have focused on exogenous antioxidants, such as polyphenols, that can scavenge free radicals and/or promote endogenous antioxidant enzymes activity, and, therefore, protect cells from oxidative damage [7].

Polyphenolic compounds are well known for their antioxidative properties. By-products from coffee processing, such as spent coffee grounds, are rich in hydrophilic phenolic compounds with antioxidant capacity. In this paper, the hydrophilic extract from SCG was obtained and its TPC, phenolic profile and antioxidant capacity were determined. The total phenolic content of the hydrophilic extract from SCG was 892.67 ± 59.12 mg GAE/100 g dry weight. Similarly, Mariotti-Celis et al. [22] reported a TPC of 821 mg GAE/100 g dry weight in extracts from SCG obtained by hot pressurized liquid extraction, using hot water and a temperature of 60 °C. Furthermore, Ramón-Gonçalves et al. [23] reported a TPC of 900 ± 200 mg GAE/100 g dry weight in extracts of SCG. Other studies have been carried out to determine the TPC of SCG, and values varied from 1754 ± 26 mg GAE/100 g dry weight [24] to 73,030 ± 20 mg GAE/100 g dry weight [25]. The differences in the TPC from these and our study might be due to the extraction conditions (solvent, temperature, time), variety of the beans, conditions of the roasting processes, among other factors [26].

Quinic acid was the main acid found in the extracts from SCG, followed by ferulic acid and caffeic acid (Table 1). In coffee residues, the quinic acid is usually esterified to caffeic, ferulic or *p*-coumaric acids to form the chlorogenic acids: caffeoylquinic acids, feruloylquinic acids and *p-*coumaroylquinic acids, respectively [27,28]. As previously reported [29], these are the most abundant phenolic acids in SCG and might represent up to 98% of its TPC [27]. In our study, the sum of the quinic, ferulic, caffeic and coumaric acids represented 82.05 ± 6.87% of the TPC of the extracts from SCG, which is in accordance with Martini et al. [27].

The antioxidant capacities of extracts from SCG were measured in terms of radical scavenging activity [30]. Roughly, it can be said that antioxidant assays used in this paper measured the antioxidant capacity of the extract from SCG by either single electron transfer (DPPH and FRAP) or hydrogen atom transfer (ORAC) [31]. It was found that the DPPH and ORAC values of the extracts from SCG were superior to the FRAP values (Table 2).

Electrochemical studies of hydroxycinnamic acids, such as caffeic acid, ferulic acid and *p-*coumaric acid, have demonstrated that in exerting its antioxidant effect, an electron transfer mechanism might occur at the same time as the hydrogen abstraction [32]. It is important to mention that due to the different chemistries related to each other method, it is not possible to compare them [33]. Nevertheless, these assays are a good indicator of the antioxidant potential of the compounds present in the extracts of SCG.

**Figure 4:**
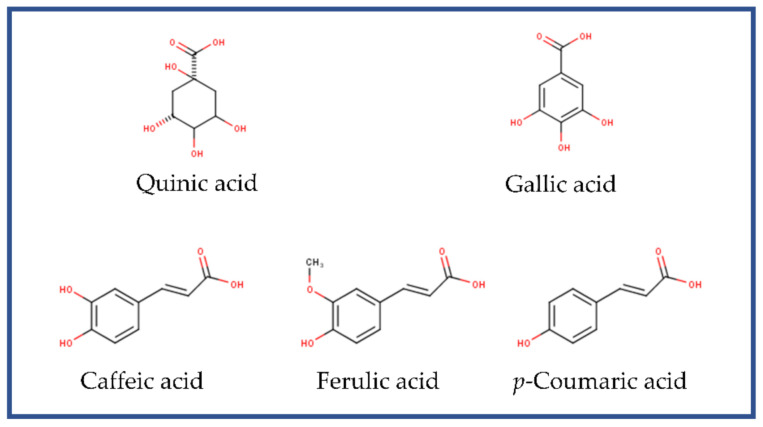
Basic chemical structure of the main compounds identified in the hydrophilic extracts from spent coffee grounds.

The antioxidant capacities of phenolic acids and their esters with quinic acid are related to the number of hydroxyl (-OH) groups in the molecule and their position [34]. Caffeic acid, ferulic acid and *p-*coumaric acid, and therefore, their respective chlorogenic acids, possess -OH groups on their aromatic rings (Figure 4). Furthermore, the pyrogallol moiety in the chemical structure of gallic acid (Figure 4) offers the phenoxyl radical a higher resonance stabilization and, therefore, higher antioxidant ability [32]. The structural characteristics of the main compounds found in the extracts of SCG in this study were used to determine their antioxidative effects, which showed that these extracts is promising candidates to evaluate their protective effects against oxidative stress.

Prior to applying the extracts from SCG, an MTT assay was performed to determine the concentration of extracts from SCG that were safe to use without exerting cytotoxic activity on the brain cells from *C. viridis*. It was observed that extract concentrations below 467.31 µg/mL maintained 80% of the cell viability, manifesting non-cytotoxic effects (Figure 1). Therefore, concentrations below 460 µg/mL were chosen to evaluate the antioxidant effect of the polyphenolic extracts from SCG against H_2_O_2_-induced oxidative stress in *C. viridis* brain cells.

H_2_O_2_ as an inducer of oxidative stress in cellular models is a very common in vitro procedure [35]. In the presence of reactive transition metal ions (such as Fe^2+^ and Cu^1+^), the H_2_O_2_ can easily degrade to produce the hydroxyl radical and initiate lipid peroxidation [36]. Secondary oxidation products are generated during the peroxidation of polyunsaturated fatty acids, namely 4-hydroxynonenal and MDA [36]. The MDA is the major reactive aldehyde produced in the peroxidation and it is, therefore, a common and suitable indicator of cellular oxidative damage [37]. In this study, it was observed that when *C. viridis* brain cells were exposed to 100 µM H_2_O_2_, MDA levels significantly increased compared to the non-exposed cells (Figure 2). This is indicative of the oxidative stress conditions in the cells. Moreover, pretreated cells with the polyphenolic extracts from SCG at 230 µg/mL and 460 µg/mL showed reduced MDA levels compared with the brain cells treated with H_2_O_2_. Previous studies showed that when PC12 cells were treated with phenolic ethanolic extracts (12.5–50 µg/mL) from *Garcinia xanthochymus* prior to the exposure to 400 µM H_2_O_2_, the MDA levels were significantly lowered [8]. Furthermore, Jiang et al. [38] reported that MDA levels in SH-SY5Y neuroblastoma cells were significantly increased after cells were exposed to 400 µM H_2_O_2_. When SH-SY5Y cells were pretreated with a caffeoylquinic acid derivative (15 and 30 µM) prior to the H_2_O_2_ exposure, the MDA levels were significantly reduced in a dose-dependent manner [39].

It has been mentioned that antioxidants such as phenolic compounds might detain or retard the lipid peroxidation reaction chain by scavenging radical species that might induce peroxidation, chelating metal ions (such as Fe^2+^ and Cu^1+^), quenching or reducing O_2_^−^, and breaking the autoxidative chain reaction [40]. The chemical properties of the phenolic compounds found in the extracts from SCG that are more likely to be involved in the chain-breaker reaction are the donation of an electron to the radical species, neutralization of such radicals and themselves becoming stable/less reactive radicals [34].

Hydrogen peroxide is a strong oxidant that diffuses easily either across membrane cells or by translocation by aquaporins (peroxiporins) [41]. It is known that at physiological levels, the H_2_O_2_ functions as a signaling molecule by upregulating several transcription factors and increasing their rate of translation to the nucleus; such is the case of AP-1, HIF-1α, Nrf2 and NFκB, among others [42]. Nrf2, the factor-erythroid 2-related factor, is associated with the upregulation of gene expression of antioxidant enzymes, such as SOD, CAT and GPx [4]. Nevertheless, it has been demonstrated that when neuronal cells are subjected to oxidative damage, such as in exposure to high H_2_O_2_ concentrations, the inhibition of the translocation of Nrf2 occurs [43], therefore affecting the gene expression of antioxidant enzymes. In this study, when brain cells from *C. viridis* were exposed to 100 µM H_2_O_2_, a significant reduction in the activity of SOD was observed (Figure 3a) compared to the control (not exposed). Even though CAT levels were also reduced (Figure 3b), this change was not significant. The decrease in the activity of these antioxidant enzymes is probably associated with the activation of GSK-3β (glycogen synthase kinase 3β) by the exposure of the cells to 100 µM H_2_O_2_, which causes the arrest of Nrf2 in the cytoplasm, therefore inhibiting its translocation to the nucleus, and the subsequent SOD and CAT gene expression [43].

The enzymes SOD and CAT play a key role in preventing cellular oxidative damage. SOD dismutates the superoxide ion (O_2_^−^) to H_2_O_2_ and oxygen, while CAT decomposes H_2_O_2_ into H_2_O and oxygen to protect cells from oxidative stress [4]. Therefore, the SOD and CAT activities were measured in this study as indicative of the enzymatic antioxidant defense system in the cell brains during oxidative stress. In the present work, a significant increase of both SOD and CAT activities was observed in the H_2_O_2_-exposed *C. viridis* brain cells pretreated with SCG at 230 µg/mL and 460 µg/mL (Figure 3). Similarly, as found in this study, Taqvi et al. [44] demonstrated that vanillic acid, a phenolic acid, exerts antioxidant effects against H_2_O_2_-induced oxidative stress in D.Mel-2 cells. Cells exposed to H_2_O_2_ significantly increased lipid peroxidation and reduced activity of SOD, CAT and GPx. D.Mel-2 cells treated with vanillic acid (0.25%) reduced lipid peroxidation and increased the activity of the antioxidant enzymes [44].

As mentioned before, translocation of the transcription factor Nrf2 from the cytosol to the nucleus of cells is a key step in the regulation of expression of the antioxidant enzymes SOD, CAT and GPx, which enable the adaptation of cells to oxidative stress [45]. Hydrogen peroxide avoids the latter and, therefore, reduces the expression of SOD and CAT [43]. The 5-*O*-caffeoylquinic acid, a caffeoylquinic acid derivative found in coffee, participates as a modulator of the Nrf2 translocation to the nucleus. It was found that this caffeoylquinic acid at concentrations from one µM to 200 µM reduced the cytosolic fraction of Nrf2 while increasing the nuclear fraction of Nrf2 in a dose-dependent manner in HT29 cells [46], which might promote the expression of antioxidant defense system enzymes. In this study, the significant increase in the activity of SOD and CAT by the extracts from SCG might be related to the ability of the phenolic components present in the extracts, and their ability to facilitate the Nrf2 nuclear translocation.

To our knowledge, this is the first report about the protective effect of extracts from the spent coffee ground against H_2_O_2_-induced oxidative stress in brain cells of *C. viridis*. In conclusion, the results of the present study demonstrated that extracts from spent coffee grounds could protect *C. viridis* brain cells from H_2_O_2_-induced oxidative damage. The protective ability is mediated by the activation of the antioxidant enzymes, SOD and CAT, and by reducing the lipid peroxidation. Therefore, we suggest polyphenolic extracts from spent coffee ground as a promising additive or supplement to reduce oxidative damage. Furthermore, research is needed in order to determine safe concentrations of extracts from SCG that exert their antioxidant protective effect in vivo, to maintain or even improve fish health, and additionally to evaluate the bioavailability and the possible interactions between the phenolic compounds of SCG extracts and the components of several food matrices used in aquaculture.

## 4. Materials and Methods

### 4.1. Collection of Spent Coffe Grounds (SCG) and Preparation of the Polyphenolic Extracts

The collection of samples was carried out from June to September 2019. Samples obtained were mixed and separated in three batches (replicates). The SCG were obtained from a well-known commercial coffee shop in Mazatlán, Sinaloa, México. According to the manager of the shop, the variety of coffee was Arabica. The SCG were dried to a constant weight in a forced-air convection drying oven at 35–37 °C for 18–24 h [47]. Subsequently, the dried materials were grounded to reduce the particle size using a GX4100 coffee grinder (KRUPS, Solingen, Germany) until a fine powder was obtained.

One g of powder from SCG was mixed with four mL of ultrapure water. Then, the mix was stirred at 55–57 °C and 100 rpm for 72 min [48]. The mixture was sonicated using a Branson 3510 ultrasonic bath (Branson Ultrasonics Corporation, Brookfield, CT, USA) for 15 min before and after the stirring. Then, the mixture was centrifuged using an Allegra X-30R centrifuge (Beckman Coulter, Brea, CA, USA) at 6500 rpm for 15 min. The supernatant, which contained the polyphenolic compounds, was immediately used for analysis. Extracts were prepared in triplicate.

### 4.2. Total Phenolic Content (TPC) Determination

Total phenolic content determination by Folin–Ciocalteu assay, was performed according to Swain and Hillis [49] with a few modifications. Briefly, 10 µL of the SCG extracts were mixed with 230 µL of distilled water and 10 µL of Folin–Ciocalteu reagent in a 96-well microplate. The mixture was incubated at 25 °C for 3 min. Lastly, 25 µL of 4 N Na_2_CO_3_ was added. The mixture was incubated for 2 h in the dark at 25 °C. After incubation, the absorbance at 750 nm was registered using an EPOCH 2NS microplate spectrophotometer (BioTek Instruments, Inc., Winooski, VT, USA). The calculations were completed using a gallic acid standard curve (from 0 to 0.4 mg/mL) and the results were expressed as milligrams of gallic acid equivalents per 100 g of dry sample (mg GAE/100 g). Measurements were made in triplicate (*n* = 3).

### 4.3. Quantification of Individual Phenolic Compounds in Spent Coffee Grounds by Ultraperformance Liquid Chromatography (UPLC)

The quantification of phenolic compounds in the extracts from SCG was determined through UPLC using an ACQUITY UPLC H-Class PLUS System (Waters Corporation, Milford, MA, USA) that used a Waters UPLC Pump (QSM) separation module (Milford, MA, USA) equipped with an autoinjector flow-through needle and a Waters PDA eλ photodiode array detector. The separation of the phenolic compounds in the extract was achieved using an ACQUITY UPLC^®®^ BEH C18 column (1.7 µm × 2.1 mm × 100 mm) maintained at 50 °C, at a flow rate of 0.3 mL/min. The mobile phase consisted of two solvents, 0.1% formic acid (in distilled water) (A) and methanol (B). The gradient system was: 90% A-10% B (0–3 min), 70% A-30% B (3–9 min), 60% A-40% B (9–11 min), 50% A-50% B (11–12 min), 0% A-100% B (12–15 min) and 90% A-10% B (15–17 min) [50]. The UV spectra were recorded from 210 to 620 nm for peak characterization. Phenolic compounds were quantified based on the peak area of the maximum absorption wavelength. Phenolic compound content was expressed as milligram of the phenolic compound per 100 g of dry weight (mg/100 g). Standard calibration curves for each phenolic compound were elaborated and measurements were made in triplicate (*n* = 3).

### 4.4. Antioxidant Capacity of Polyphenolic Extracts from SCG

#### 4.4.1. 2,2-Diphenyl-1-picrylhydrazyl Free Radical (DPPH^•^) Scavenging Activity Assay

DPPH radical scavenging activity assay was performed according to Brand-Williams et al. [51], with slight modifications. Briefly, 20 µL of the SCG extracts were mixed with 280 μL of the DPPH reagent (100 µM in ethanol). After 30 min of incubation, the absorbance was recorded at 540 nm in an EPOCH 2NS microplate spectrophotometer (Biotek Instruments, Inc., Winooski, VT, USA). The results were calculated from a Trolox standard curve (from 0 to 400 µM) and were expressed as micromoles of Trolox equivalent per 100 g of dry sample (µmol TE/100 g). Measurements were made in triplicate (*n* = 3).

#### 4.4.2. Ferric Ion Reducing Antioxidant Power (FRAP) Assay

The FRAP assay was carried out following the procedure described by Benzie and Strain [52] with slight modifications. An aliquot of 20 μL of the SCG extract was mixed with 225 μL of FRAP reagent (300 mM sodium acetate buffer pH 3.6, 10 mM 2,4,6-tripyridyl-*s*-triazine, and 20 mM FeCl_3_·6H_2_O, at a ratio of 10:1:1, *v*:*v*:*v*). The reaction mixture was incubated at 37 °C for 30 min. The absorbance was read at 593 nm using an EPOCH 2NS microplate spectrophotometer (Biotek Instruments, Inc., Winooski, VT, USA). The calculations were achieved using a Trolox standard curve (from 0 to 500 µM) and the results were expressed as micromoles of Trolox equivalent per 100 g of dry sample (µmol TE/100 g). Measurements were made in triplicate (*n* = 3).

#### 4.4.3. Oxygen Radical Absorbance Capacity (ORAC) Assay

For the ORAC assay [53], an aliquot of 25 µL of the SCG extract, Trolox standards (from 6.25 to 125 µM) or blank solution (75 mM phosphate buffer, pH 7.4) were added to each well in a 96-well microplate. Then, 200 µL of 0.106 µM fluorescein followed by 75 μL of 800 mM 2,2′-azobis(2-methylpropionamidine)-dihydrochloride (AAPH) were automatically injected into each well. Fluorescence was detected every 70 s for 70 min, at 485 nm (excitation) and 580 nm (emission) using a Biotek Synergy HT microplate reader (Biotek Instruments, Inc., Winooski, VT, USA). Results were determined as the differences between the area under the curve of the sample and the blank and the Trolox curve. Results were expressed as micromoles of Trolox equivalent per gram of dry sample (µmol TE/g). Measurements were made in triplicate (*n* = 3).

### 4.5. Cell Culture

MTT Formazan (1-(4,5-dimethylthiazol-2-yl)-3,5-diphenylformazan, thiazolyl blue formazan (CAS Number: 57360-69-7) and bovine serum albumin (BSA) were purchased from Sigma Chemical (St. Louis, MO, USA). Dulbecco’s modified Eagle medium (DMEM), L-15 medium (Leibovitz), antibiotic–antimycotic (100X) and fetal bovine serum (FBS) were purchased from GIBCO BRL (Grand Island, NY, USA). Primary fish cell cultures were obtained from the cell and tissue culture laboratory of the Centro de Investigación en Alimentación y Desarrollo, A. C. Mazatlán (Center for Research in Food and Development). All procedures and protocols were performed under the Official Mexican Standard (NOM-033-ZOO-1995 regulation, which refers to the humanitarian sacrifice of domestic and wild animals); the protocols are in accordance with international guidelines for the use of animals in research.

Cell cultures of *C. viridis* brain cells were maintained at 27 °C in a humidified 5% CO_2_ atmosphere with growth medium (DMEM medium containing 10% fetal bovine serum, 1% of penicillin + streptomycin, 100 U/mL of penicillin + 100 µg/mL of streptomycin) changed every other day.

### 4.6. Determination of the Non-Toxic Concentrations of Spent Coffee Ground Polyphenolic Extracts. Thiazolyl Blue Tetrazolium Bromide (MTT) Cytotoxicity Assay

The thiazolyl blue tetrazolium bromide (MTT) assay was carried out by modifying the original Mosmann [54]. Brain cells (12.5 × 10^3^) were plated in a 96-well plate and allowed to adhere and spread for 24 h. Polyphenolic extracts from the spent coffee ground were added in varying concentrations (0–2000 µg/mL), and the cells were cultured for 24 h at 27 °C. MTT solution (5 mg/mL, in sterile PBS) was added to each well, and the cultures were incubated for an additional 4 h. After incubation, MTT was removed and crystals formed were solubilized with acidified isopropanol. The absorbance at 570 nm was determined in each well with a 96-well plate reader. The growth of treated cells was compared with that of untreated cells. Each polyphenolic extract concentration was examined in four replicate wells, and the percentage of viable cells was determined [55].

### 4.7. H_2_O_2_-Induced Oxidative Stress Trial

To examine the effect of the polyphenol extracts from SCG on antioxidant enzymes activity and lipid peroxidation, cells were seeded at a density of 0.5 × 10^6^ cells per well in 6-well plates. Cells were pretreated for 24 h with the SCG extract, at 115, 230 and 460 µg/mL. Afterward, 100 µM H_2_O_2_ [56,57] was added to induce the oxidative stress, and cells were incubated for one hour.

Treatments are represented as follows:

- C: control, non-treated cells.
- C+: cells treated with 100 µM H_2_O_2_ alone.
- SCG: cells pretreated with the spent coffee ground polyphenolic extracts at 115, 230 and 460 µg/mL, and treated with 100 µM H_2_O_2_.

Following incubation, the cells were washed with PBS and detached using a cold solution of PBS/0.5 M EDTA (2%) to achieve a non-enzymatic cellular dissociation. Subsequently, the cell suspension was centrifuged at 13,000× *g* for 10 min at 4 °C. The cellular pellet was recovered, resuspended in 300 µL of the extraction buffer (phosphate buffer, 50 mM, pH 7.4) and sonicated for 10 min; subsequently, the suspension was again centrifuged at 13,000× *g* for 10 min at 4 °C and the supernatant was recovered. The supernatant was used immediately for the antioxidant enzyme activity analysis and lipid peroxidation assay.

### 4.8. Assay for Lipid Peroxidation

This assay was performed according to Lizárraga-Velázquez et al. [58] and Solé et al. [59] with slight modifications. Briefly, 50 μL of the supernatant were mixed with 163 μL of 1 methyl-2-phenylindole (10.3 mM, in methanol:acetonitrile, 1:3; *v/v*), 50 μL of distilled water and 75 μL of HCl at 37%. This reaction mix was incubated at 45 °C for 40 min. Next, the reaction solution was cooled on ice for 10 min and centrifuged at 3000× *g* for 15 min at 4 °C. The absorbance was recorded at 586 nm using an EPOCH 2NS microplate spectrophotometer (Biotek Instruments, Inc., Winooski, VT, USA). The calculations were performed by using a 1,1,3,3-tetramethoxypropane (Sigma Aldrich 108383-100ML) standard curve (from 0 to 12 µM) and the results were expressed as micromoles of malondialdehyde per gram of cellular pellet (μmol MDA/g pellet). Measurements were made in triplicate (*n* = 3).

### 4.9. Assay for Antioxidant Enzymes

#### 4.9.1. Superoxide Dismutase Activity

Enzymatic activity of SOD was determined using the SOD-WST kit (19160 Sigma Aldrich) following the manufacturer’s instructions. Briefly, 20 µL of the enzymatic extract (supernatant) and 200 µL of the WST solution were added to a 96-well plate and mixed by gently pipetting. Later, 20 µL of enzyme working solution was added and mixed again. The plate was incubated at 37 °C for 20 min. Absorbance was read via a 450 nm EPOCH 2NS microplate spectrophotometer (Biotek Instruments, Inc., Winooski, VT, USA) and the results were expressed as units of SOD activity per milligram of protein (U/mg protein). One unit of SOD activity was defined as the amount of enzyme that inhibits formazan formation by 50%.

#### 4.9.2. Catalase Activity

Catalase activity was determined according to Aebi [60] with slight modifications. The assay reaction mix consisted of 200 µL of 15 mM H_2_O_2_ dissolved in 100 mM phosphate buffer (pH 6.5). Subsequently, 10 µL of the supernatant was added and reaction kinetics were immediately carried out for two minutes with readings every 10 s. Readings were done at 240 nm in an EPOCH 2NS microplate spectrophotometer (Biotek Instruments, Inc., Winooski, VT, USA). The results were expressed as units of CAT activity per milligram of protein (U/mg protein). One unit of catalase activity was defined as the amount of enzyme needed to catalyze 1 mmol of H_2_O_2_ per minute at 25 °C.

#### 4.9.3. Concentration of Protein

In order to express the enzymatic activity as U/mg protein, the concentration of protein in the supernatant was determined according to Bradford [61]. Briefly, 5 µL of supernatant was mixed with 250 µL of Bradford reagent (B6916, Sigma Aldrich) and incubated for 15 min at room temperature (25 °C). Afterward, absorbance at 595 nm was recorded in an EPOCH 2NS microplate spectrophotometer (Biotek Instruments, Inc., Winooski, VT, USA). The results were calculated from a bovine serum albumin standard curve (from 0.1 to 1.4 mg/mL). Measurements were made in triplicate (*n* = 3).

### 4.10. Statistical Analysis

Data were tested for normality using the Anderson–Darling test. The rest of the data were analyzed using one-way analysis of variance (ANOVA) followed by Tukey’s HSD test using the software Minitab version 17.1 (Minitab Inc., State College, PA, USA). The results are expressed as means ± standard deviations (SD). Different letters show significant differences *(p* < 0.05).

## Figures and Tables

**Table 1:** Main phenolic compounds present in the hydrophilic extracts of spent coffee grounds.

Compound	Rt *	Concentration (mg/100 g D.W.)	Contribution (%)
Quinic acid	0.9	656.43 ± 47.92	73.59 ± 5.37
Gallic acid	1.59	6.63 ± 0.80	0.74 ± 0.09
Caffeic acid	4.19	36.79 ± 10.06	4.12 ± 1.13
Coumaric acid	5.09	1.28 ± 0.33	0.14 ± 0.04
Ferulic acid	5.53	37.37 ± 3.64	4.19 ± 0.41

* Rt = retention time (min) (Supplementary Materials Files S1–S5).

**Table 2:** Antioxidant capacity of hydrophilic extracts from spent coffee grounds.

DPPH	FRAP	ORAC
3672.39 ± 602.12 ^1^	428.29 ± 17.53 ^1^	2417.42 ± 149.37 ^1^

^1^ µmol TE/100 g.

## Data Availability

The data presented in this study is available, upon reasonable request, from the corresponding authors.

## Supplementary Materials

The following are available online at https://www.mdpi.com/article/10.3390/molecules26206195/s1, File S1: Calibration curve, correlation coefficients and retention time of quinic acid determined by UPLC-PDA; File S2: Calibration curve, correlation coefficients and retention time of gallic acid determined by UPLC-PDA; File S3: Calibration curve, correlation coefficients and retention time of caffeic acid determined by UPLC-PDA; File S4: Calibration curve, correlation coefficients and retention time of coumaric acid determined by UPLC-PDA; File S5: Calibration curve, correlation coefficients and retention time of ferulic acid determined by UPLC-PDA.

## Sample Availability

Samples of the compounds are available from the authors.
