# Supplementary material for: Polyphenolic Extracts from Spent Coffee Grounds Prevent H2O2-Induced Oxidative Stress in Centropomus viridis Brain Cells
Source: Molecules. 2021 Oct 14;26(20):6195. doi: 10.3390/molecules26206195 (PMC8540615; doi:10.3390/molecules26206195)
Supplement: Supplementary file 1 [file molecules-26-06195-s001.zip › File S4_Cafe 1_cumarico.pdf]

Dataset: Untitled

Last Altered: Friday, May 14, 2021 22:47:41 Mountain Daylight Time (Mexico)
Printed: Friday, May 14, 2021 22:47:54 Mountain Daylight Time (Mexico)

Method: C:\MassLynx\waters1.PRO\MethDB\Mayo cumarico 3.mdb 14 May 2021 15:21:28
Calibration: C:\MassLynx\waters1.PRO\CurveDB\New folder\Curva_cumarico_mayo_3.cdb 14 May 2021 22:47:41

Compound name: ac. cumarico

	#	Name	Type	RT	Area	Response	ug/mL	%Dev
1	1	cafe-004	Analyte	5.09	160.255	160.255	0.071	

Compound name: ac. cumarico
Coefficient of Determination: R^2 = 0.988514
Calibration curve: 2260.45 * x
Response type: External Std, Area
Curve type: Linear, Origin: Force, Weighting: 1/x, Axis trans: None

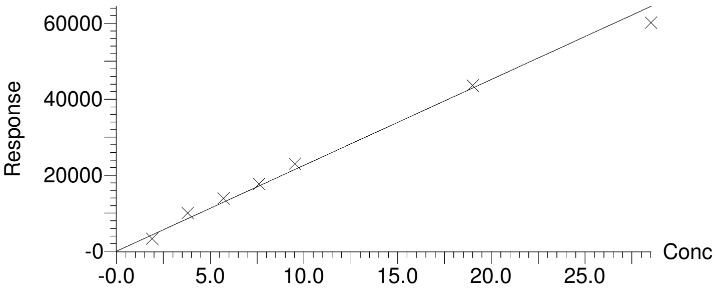

Dataset: Untitled

Last Altered: Friday, May 14, 2021 22:47:41 Mountain Daylight Time (Mexico)
Printed: Friday, May 14, 2021 22:47:54 Mountain Daylight Time (Mexico)

Method: C:\MassLynx\waters1.PRO\MethDB\Mayo cumarico 3.mdb 14 May 2021 15:21:28
Calibration: C:\MassLynx\waters1.PRO\CurveDB\New folder\Curva_cumarico_mayo_3.cdb 14 May 2021 22:47:41

Compound name: ac. cumarico
Coefficient of Determination: R^2 = 0.988514
Calibration curve: 2260.45 * x
Response type: External Std, Area
Curve type: Linear, Origin: Force, Weighting: 1/x, Axis trans: None

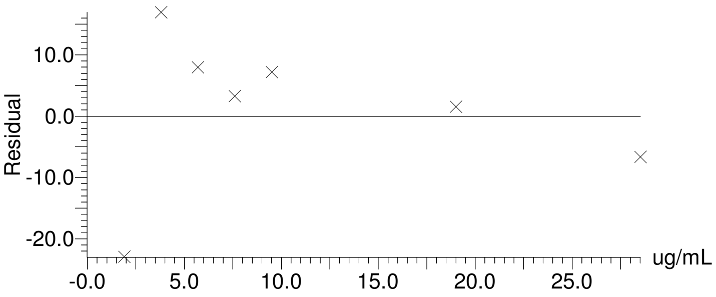

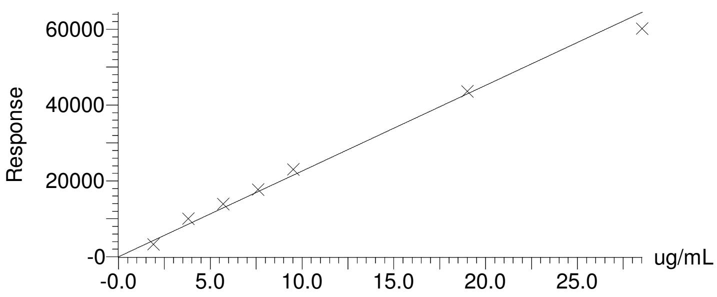

Dataset: Untitled

Last Altered: Friday, May 14, 2021 22:47:41 Mountain Daylight Time (Mexico)
Printed: Friday, May 14, 2021 22:47:54 Mountain Daylight Time (Mexico)

Method: C:\MassLynx\waters1.PRO\MethDB\Mayo cumarico 3.mdb 14 May 2021 15:21:28
Calibration: C:\MassLynx\waters1.PRO\CurveDB\New folder\Curva_cumarico_mayo_3.cdb 14 May 2021 22:47:41

Name: cafe-004, Date: 14-May-2021, Time: 15:03:41, ID: , Description: 1

ac. cumarico

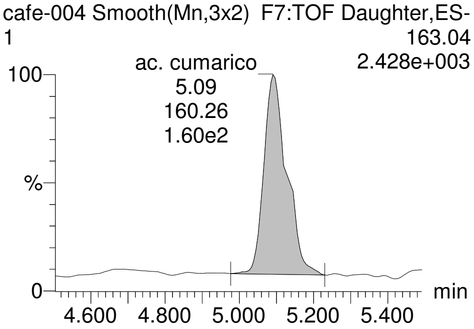

ID	Name	Trace	RT	Area	ug/mL
	ac. cumarico	163.04	5.09	160.255	0.071
