# Supplementary material for: Polyphenolic Extracts from Spent Coffee Grounds Prevent H2O2-Induced Oxidative Stress in Centropomus viridis Brain Cells
Source: Molecules. 2021 Oct 14;26(20):6195. doi: 10.3390/molecules26206195 (PMC8540615; doi:10.3390/molecules26206195)
Supplement: Supplementary file 1 [file molecules-26-06195-s001.zip › File S5_Cafe 1_ferulico.pdf]

Dataset: Untitled

Last Altered: Friday, May 14, 2021 22:48:54 Mountain Daylight Time (Mexico)
Printed: Friday, May 14, 2021 22:49:15 Mountain Daylight Time (Mexico)

Method: C:\MassLynx\waters1.PRO\MethDB\Mayo ferulico 3 tiempo.mdb 14 May 2021 15:21:58
Calibration: C:\MassLynx\waters1.PRO\CurveDB\New folder\Curva_ferulico_mayo_tiempo_3.cdb 14 May 2021 15:17:25

Compound name: ac ferulico

	#	Name	Type	RT	Area	Response	ug/mL	%Dev
1	1	cafe-004	Analyte	5.53	1048.208	1048.208	2.138	

Compound name: ac ferulico
Correlation coefficient: r = 0.989711, r^2 = 0.979527
Calibration curve: 486.244 * x + 8.6903
Response type: External Std, Area
Curve type: Linear, Origin: Exclude, Weighting: 1/x, Axis trans: None

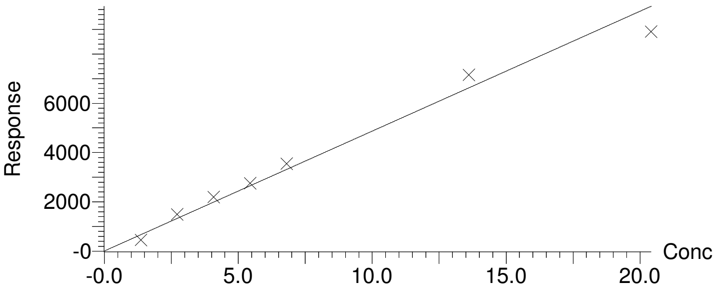

Dataset: Untitled

Last Altered: Friday, May 14, 2021 22:48:54 Mountain Daylight Time (Mexico)
Printed: Friday, May 14, 2021 22:49:15 Mountain Daylight Time (Mexico)

Method: C:\MassLynx\waters1.PRO\MethDB\Mayo ferulico 3 tiempo.mdb 14 May 2021 15:21:58
Calibration: C:\MassLynx\waters1.PRO\CurveDB\New folder\Curva_ferulico_mayo_tiempo_3.cdb 14 May 2021 15:17:25

Compound name: ac ferulico
Correlation coefficient: r = 0.989711, r^2 = 0.979527
Calibration curve: 486.244 * x + 8.6903
Response type: External Std, Area
Curve type: Linear, Origin: Exclude, Weighting: 1/x, Axis trans: None

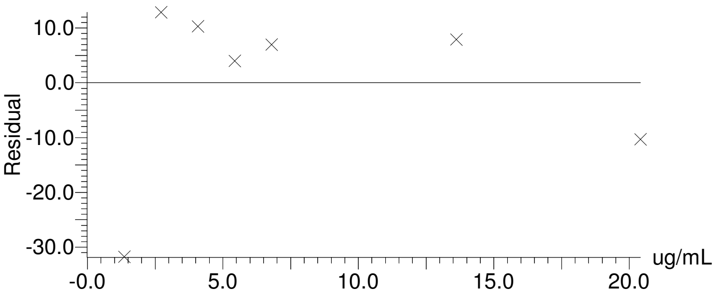

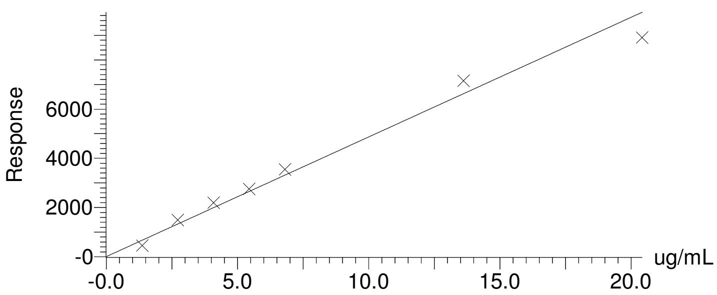

Dataset: Untitled

Last Altered: Friday, May 14, 2021 22:48:54 Mountain Daylight Time (Mexico)
Printed: Friday, May 14, 2021 22:49:15 Mountain Daylight Time (Mexico)

Method: C:\MassLynx\waters1.PRO\MethDB\Mayo ferulico 3 tiempo.mdb 14 May 2021 15:21:58
Calibration: C:\MassLynx\waters1.PRO\CurveDB\New folder\Curva_ferulico_mayo_tiempo_3.cdb 14 May 2021 15:17:25

Name: cafe-004, Date: 14-May-2021, Time: 15:03:41, ID: , Description: 1

ac ferulico

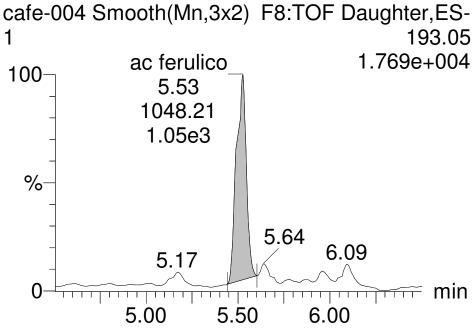

ID	Name	Trace	RT	Area	ug/mL
	ac ferulico	193.05	5.53	1048.208	2.138
