# Supplementary material for: Polyphenolic Extracts from Spent Coffee Grounds Prevent H2O2-Induced Oxidative Stress in Centropomus viridis Brain Cells
Source: Molecules. 2021 Oct 14;26(20):6195. doi: 10.3390/molecules26206195 (PMC8540615; doi:10.3390/molecules26206195)
Supplement: Supplementary file 1 [file molecules-26-06195-s001.zip › File S3_Cafe 1_cafeico.pdf]

Dataset: Untitled

Last Altered: Friday, May 14, 2021 22:46:36 Mountain Daylight Time (Mexico)
Printed: Friday, May 14, 2021 22:46:48 Mountain Daylight Time (Mexico)

Method: C:\MassLynx\waters1.PRO\MethDB\Mayo cafeico 3.mdb 14 May 2021 15:20:57
Calibration: C:\MassLynx\waters1.PRO\CurveDB\New folder\Curva_cafeico_mayo_3.cdb 14 May 2021 15:15:15

Compound name: ac. cafeico

	#	Name	Type	RT	Area	Response	ug/mL	%Dev
1	1	cafe-004	Analyte	4.19	11330.568	11330.568	2.024	

Compound name: ac. cafeico
Correlation coefficient: r = 0.994236, r^2 = 0.988504
Calibration curve: 4724.73 * x + 1768.68
Response type: External Std, Area
Curve type: Linear, Origin: Exclude, Weighting: 1/x, Axis trans: None

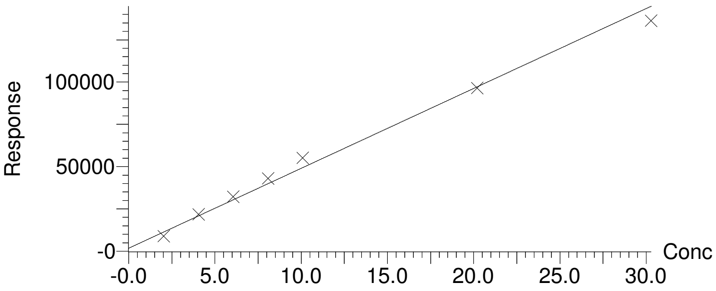

Dataset: Untitled

Last Altered: Friday, May 14, 2021 22:46:36 Mountain Daylight Time (Mexico)
Printed: Friday, May 14, 2021 22:46:48 Mountain Daylight Time (Mexico)

Method: C:\MassLynx\waters1.PRO\MethDB\Mayo cafeico 3.mdb 14 May 2021 15:20:57
Calibration: C:\MassLynx\waters1.PRO\CurveDB\New folder\Curva_cafeico_mayo_3.cdb 14 May 2021 15:15:15

Compound name: ac. cafeico
Correlation coefficient: r = 0.994236, r^2 = 0.988504
Calibration curve: 4724.73 * x + 1768.68
Response type: External Std, Area
Curve type: Linear, Origin: Exclude, Weighting: 1/x, Axis trans: None

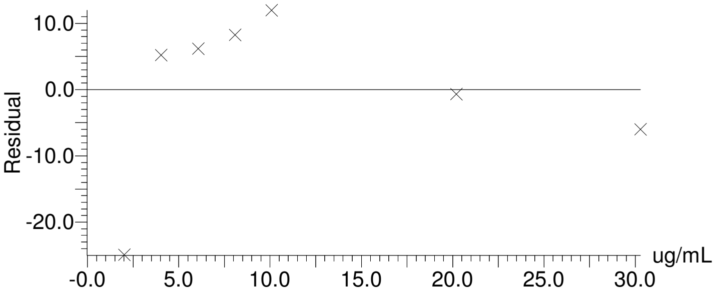

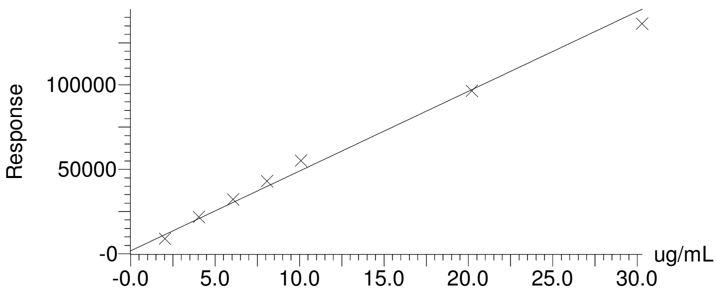

Dataset: Untitled

Last Altered: Friday, May 14, 2021 22:46:36 Mountain Daylight Time (Mexico)
Printed: Friday, May 14, 2021 22:46:48 Mountain Daylight Time (Mexico)

Method: C:\MassLynx\waters1.PRO\MethDB\Mayo cafeico 3.mdb 14 May 2021 15:20:57
Calibration: C:\MassLynx\waters1.PRO\CurveDB\New folder\Curva_cafeico_mayo_3.cdb 14 May 2021 15:15:15

Name: cafe-004, Date: 14-May-2021, Time: 15:03:41, ID: , Description: 1

ac. cafeico

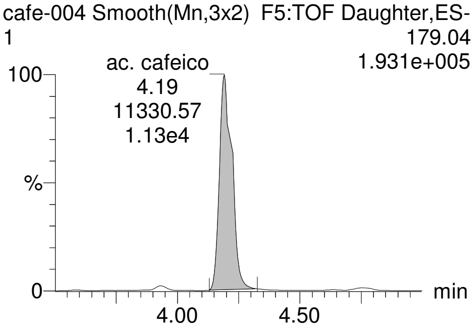

ID	Name	Trace	RT	Area	ug/mL
	ac. cafeico	179.04	4.19	11330.568	2.024
