# Supplementary material for: Polyphenolic Extracts from Spent Coffee Grounds Prevent H2O2-Induced Oxidative Stress in Centropomus viridis Brain Cells
Source: Molecules. 2021 Oct 14;26(20):6195. doi: 10.3390/molecules26206195 (PMC8540615; doi:10.3390/molecules26206195)
Supplement: Supplementary file 1 [file molecules-26-06195-s001.zip › File S2_Cafe 1_galico.pdf]

Dataset: Untitled

Last Altered: Friday, May 14, 2021 22:49:55 Mountain Daylight Time (Mexico)
Printed: Friday, May 14, 2021 22:50:18 Mountain Daylight Time (Mexico)

Method: C:\MassLynx\waters1.PRO\MethDB\Mayo galico 3.mdb 14 May 2021 15:22:29
Calibration: C:\MassLynx\waters1.PRO\CurveDB\New folder\Curva_galico_mayo_3.cdb 14 May 2021 15:18:53

Compound name: ac. galico

	#	Name	Type	RT	Area	Response	ug/mL	%Dev
1	1	cafe-004	Analyte	1.59	639.337	639.337	0.434	

Compound name: ac. galico
Correlation coefficient: r = 0.998427, r^2 = 0.996857
Calibration curve: 2699.53 * x + -532.521
Response type: External Std, Area
Curve type: Linear, Origin: Exclude, Weighting: 1/x, Axis trans: None

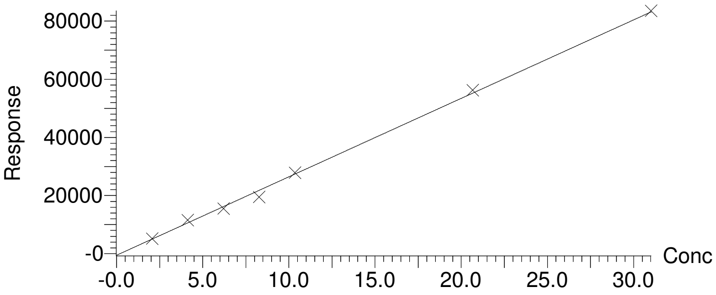

Dataset: Untitled

Last Altered: Friday, May 14, 2021 22:49:55 Mountain Daylight Time (Mexico)
Printed: Friday, May 14, 2021 22:50:18 Mountain Daylight Time (Mexico)

Method: C:\MassLynx\waters1.PRO\MethDB\Mayo galico 3.mdb 14 May 2021 15:22:29
Calibration: C:\MassLynx\waters1.PRO\CurveDB\New folder\Curva_galico_mayo_3.cdb 14 May 2021 15:18:53

Compound name: ac. galico
Correlation coefficient: r = 0.998427, r^2 = 0.996857
Calibration curve: 2699.53 * x + -532.521
Response type: External Std, Area
Curve type: Linear, Origin: Exclude, Weighting: 1/x, Axis trans: None

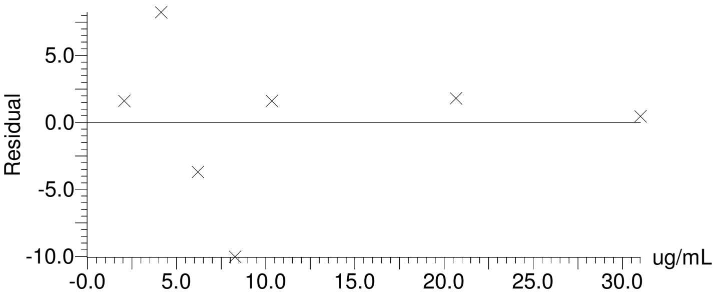

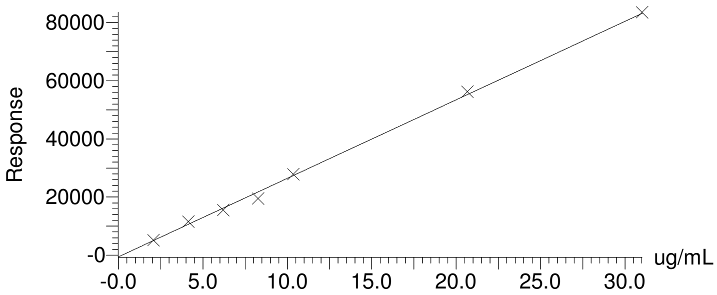

Dataset: Untitled

Last Altered: Friday, May 14, 2021 22:49:55 Mountain Daylight Time (Mexico)
Printed: Friday, May 14, 2021 22:50:18 Mountain Daylight Time (Mexico)

Method: C:\MassLynx\waters1.PRO\MethDB\Mayo galico 3.mdb 14 May 2021 15:22:29
Calibration: C:\MassLynx\waters1.PRO\CurveDB\New folder\Curva_galico_mayo_3.cdb 14 May 2021 15:18:53

Name: cafe-004, Date: 14-May-2021, Time: 15:03:41, ID: , Description: 1

ac. galico

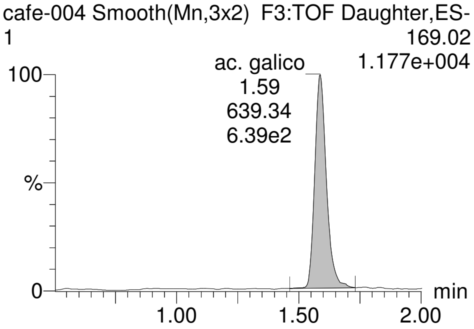

ID	Name	Trace	RT	Area	ug/mL
	ac. galico	169.02	1.59	639.337	0.434
